# Implementation of a Real-Time Brain-to-Brain Synchrony Estimation Algorithm for Neuroeducation Applications

**DOI:** 10.3390/s24061776

**Published:** 2024-03-09

**Authors:** Axel A. Mendoza-Armenta, Paula Blanco-Téllez, Adaliz G. García-Alcántar, Ivet Ceballos-González, María A. Hernández-Mustieles, Ricardo A. Ramírez-Mendoza, Jorge de J. Lozoya-Santos, Mauricio A. Ramírez-Moreno

**Affiliations:** School of Engineering and Sciences, Mechatronics Department, Tecnologico de Monterrey, Monterrey 64700, Mexico; axel.mendoza@tec.mx (A.A.M.-A.); a01378323@tec.mx (P.B.-T.); a01067645@tec.mx (A.G.G.-A.); a01066960@tec.mx (I.C.-G.); a00827005@exatec.tec.mx (M.A.H.-M.); ricardo.ramirez@tec.mx (R.A.R.-M.); jorge.lozoya@tec.mx (J.d.J.L.-S.)

**Keywords:** EEG, bispectrum, brain-to-brain synchronization, real-time algorithm, physiological signals, Python

## Abstract

This study centers on creating a real-time algorithm to estimate brain-to-brain synchronization during social interactions, specifically in collaborative and competitive scenarios. This type of algorithm can provide useful information in the educational context, for instance, during teacher–student or student–student interactions. Positioned within the context of neuroeducation and hyperscanning, this research addresses the need for biomarkers as metrics for feedback, a missing element in current teaching methods. Implementing the bispectrum technique with multiprocessing functions in Python, the algorithm effectively processes electroencephalography signals and estimates brain-to-brain synchronization between pairs of subjects during (competitive and collaborative) activities that imply specific cognitive processes. Noteworthy differences, such as higher bispectrum values in collaborative tasks compared to competitive ones, emerge with reliability, showing a total of 33.75% of significant results validated through a statistical test. While acknowledging progress, this study identifies areas of opportunity, including embedded operations, wider testing, and improved result visualization. Beyond academia, the algorithm’s utility extends to classrooms, industries, and any setting involving human interactions. Moreover, the presented algorithm is shared openly, to facilitate implementations by other researchers, and is easily adjustable to other electroencephalography devices. This research not only bridges a technological gap but also contributes insights into the importance of interactions in educational contexts.

## 1. Introduction

Human beings naturally employ cognitive processes and establish social interactions during interpersonal encounters. Memory, language, and attention play fundamental roles in our personal development within this framework [1]. In this context, learning is an essential task that requires research to further understand the changes in brain activity that take place. The education system relies on a dynamic teaching system that thrives on active feedback and continuous improvement of the interaction between students and their teachers, which gives rise to meaningful social connections that demand high cognitive engagement [2]. Consequently, assessing the level of brain synchronization between individuals could serve as a valuable metric to distinguish the effectiveness of academic interactions.

In order to generate intelligent neuroeducational tools, real-time algorithms are needed to monitor and track brain synchrony levels during a given activity, providing instantaneous feedback to take action depending on the observed patterns in various subjects. However, most hyperscanning studies rely on offline analysis, rather than real-time applications [1]. Consequently, it is necessary to develop an accessible and user-friendly system that serves as an assistive technology for educational institutions to enhance their teaching models, via neural synchrony metrics. The development of algorithms for the estimation of real-time brain synchronization would facilitate the evaluation of various learning modalities and therefore provide evidence for their improvement. It would enable feedback on the instructor–student relationship and assess how this level of synchronization influences academic performance. Additionally, this information can be utilized to implement changes aimed at improving the teaching models.

The aim of this work is to present the implementation of a real-time algorithm for estimating brain synchronization using wireless EEG. The architecture for synchronized data acquisition is based on the Advanced Learner Assistance System (ALAS) [3], adding new real-time processing capabilities. The brain-to-brain (B2B) feature extraction follows the methods presented by Ramírez-Moreno et al. [4]. The algorithm was built under the open-access framework Python, facilitating its dissemination and sharing, and it holds significant impact on the neuroeducation field. It would allow for more efficient and personalized teaching and learning methods, real-time monitoring of interaction quality, improved collaboration and teamwork inside the classroom, and a deeper understanding of shared brain dynamics during social interactions [5].

An evaluation of the algorithm was performed with subjects engaging in collaborative and competitive tasks. In this way, two different kinds of activities can influence the results. Social interactions are triggered by assembling a puzzle as a team, which is entirely different from playing dominoes, a game of dexterity with personal interests in a one-versus-one match. The results are encouraging; applying a Wilcoxon rank sum test, significant differences were found in B2B synchrony estimation while subjects performed collaborative tasks, consistent with what has been reported in the literature.

The presented algorithm is the first architecture capable of pre-processing and applying bispectral analysis to two different EEG signals while they are being extracted. Considering that a variety of techniques and methods have been tested for real-time EEG pre-processing, with significant impact on the diagnosis of diseases and other emotion-based applications [6], this implementation is the first of its kind, with an analysis centered on students’ social interactions for potential applications inside classrooms. Even though inter-brain synchrony has been compared in collaborative and competitive scenarios in other investigations [7], instantaneous feedback has not been proposed. This study opens the possibility for real-time interpretations and a high impact on neuroeducation if further code improvements are developed with more refined analysis techniques.

### Bibliographic Review

Neuroeducation, also known as educational neuroscience, is a discipline that seeks to renew learning and instruction through neuroengineering. Considering that learning and teaching are directly related to the brain functions of each person, its application has led to revolutionary advances over the years [8]. As a result, neurocognitive programs and new studies on brain behavior have been developed, along with diagnostic tools, such as effective stimulation [9]. The constant pursuit of understanding brain functionality has made neuroeducation a determining factor in analyzing various psychological and social behaviors. This is reflected in educational settings, allowing for the measurement and evaluation of diverse attitudes, actions, and behaviors that are related to cognitive aspects and brain development in humans [10].

Within educational innovation, the use of electroencephalography (EEG) for the monitoring of brain signals during learning tests has been proposed, as it has proven to provide a numerical understanding of cognitive processes [11]. By combining this technique with signal analysis and machine learning, it is possible to identify the spectral or temporal variables of the EEG that correlate with increased or reduced cognitive performance, including states of attention and distraction, mental fatigue, and drowsiness, amongst others [12].

Brain-to-brain synchrony, also called neural synchrony or inter-brain coupling, refers to the coordination of the brain response of at least two individuals to a stimulus and the increase in these similar activity patterns over time [13,14]. The analysis of synchronized EEG tests of multiple users, also known as hyperscanning has started to gain momentum in neuroscience, as it opens the window to studying social interactions, as well as their reflection in the dynamics of shared brain activity [15]. EEG hyperscanning refers mainly to the simultaneous and synchronized recording of EEG data among multiple participants, often while they are engaged in a shared activity. One of the challenges of hyperscanning is the need for multiple EEG systems, which restricts the feasibility of such studies but remains accessible due its relative low-cost implementation [16]. When it comes to the analysis of neurophysiological correlations of social interactions, two measures of hyperscanning can be obtained: intra-brain and inter-brain connectivity. The approach of this study analyzes inter-brain connectivity, which refers to functional connectivity between the brain regions of different individuals responsible for interpersonal coupling during social interactions. Both metrics are adequate predictors of collective educational performance [17].

Typical EEG analysis includes its decomposition into spectral features (frequency bands). These bands are interpreted based on their functional significance, which is related to specific cognitive and emotional functions or processes [17], and it can be implemented in hyperscanning studies, as well. Reported hyperscanning studies have shown the types of metrics commonly used to estimate brain synchronization, such as coherence, Phase Coherence, Phase Locking Value (PLV), Phase Locking Index (PLI), Granger Causality, correlation, Wavelet Transform Coherence (WTC), Graph Theory, and Partial Directed Coherence (PDC), and bispectrum [4].

Bispectral analysis is a technique used to analyze interactions and synchrony between pairs of signals, whether they are linear or nonlinear in the signal generation process. Due to this capability, bispectral analysis is a more suitable tool than spectral power analysis for quantifying subtle changes in brain activity [18]. Furthermore, through bispectral analysis, it is possible to capture the phase relationships between frequency components and temporal EEG data [19]. The bispectrum is a metric that provides information about the temporal, spatial, and spectral levels of pairs of signals. Unlike other metrics of brain synchronization, it is a higher-order spectrum feature that refers to the degree of temporal synchronization, phase coupling, and nonlinear interactions between any pair of signals analyzed in different frequency bands [4].

Currently, different algorithms exist to describe B2B synchronization. One innovative framework captures and analyzes cognitive responses, providing insights into learner engagement and comprehension during online courses, potentially enhancing effectiveness based on individual cognitive states [20]. Another study investigates brain synchronization during communication, focusing on speaking and listening activities and offering insights into simultaneous brain activity during interaction [21]. A systematic review revealed how the use of coherence measures helps us to understand brain synchronization within Brain–Computer Interface (BCI) research, highlighting the necessity for more controlled experiments to enhance future research outcomes [22].

Aside from the study of social interactions, hyperscanning has also been studied within the educational context [23]. When it comes to EEG, various researchers have obtained representations of synchronized brain activity in individuals while they perform an activity simultaneously [24]. Some studies have shown that when participants perform a creative group activity, neural connections intensify during moments of mutual gaze and activate the same brain regions, facilitating cognitive processes. This way, the knowledge of the participants is accelerated, which represents a cognitive improvement [5]. The implementation of brain synchrony-based tools in the educational field would provide tremendous support in the design and evaluation of learning models and teaching strategies where student–student and student–teacher interactions play an important role.

The paper is structured as follows: Section 2 provides a detailed description of the materials and methods used in this work. The results of the implementation are shown in Section 3 and are discussed in depth in Section 4. Finally, this paper concludes in Section 5 with the conclusions.

## 2. Materials and Methods

### 2.1. Software

The main objective of this investigation is to develop an algorithm that can process brain signal data (from two individuals) and determine different levels of B2B synchrony in real time. To achieve this, a Python code was developed. Python was chosen as the programming language for its simplicity, open-source nature, and flexibility with any operating system, providing a solution with great potential for future expansion and sharing.

The current implementation acquires EEG signals from two participants in parallel, performs simple pre-processing to both signals, and then generates the B2B synchrony estimations in real time. One of the advantages of the selected programming framework is that it has vast community support, which ensures a smooth development process and the possibility of further enhancements as the project evolves.

Python offers a diverse array of libraries that greatly facilitate specific tasks and processes, enabling the development of simple yet powerful code. The algorithm at hand requires several essential components, including external device connections, data processing, matrix operations, Fast Fourier Transform (FFT) calculations, and graphical display. To fulfill these requirements, the following main libraries are listed below:

- Brainflow: A multi-language library (supporting Python (3.8.10) in the environment created for the execution of the algorithm), that enables the acquisition of signals from various biosensors (mostly EEG), regardless of their commercial brand or type, refer to its Installation Instructions “https://brainflow.readthedocs.io/en/stable/SupportedBoards.html (accessed on 20 February 2023)” to see all supported softwares and their versions. It has multiple commands optimized for EEG signal analysis, like real-time processing and feature extraction.
- Multiprocessing: This package is employed for process creation, providing local and remote concurrency, and maximizing the utilization of a given machine, forcing the execution of the declared functions in the code in parallel.
- PyQtGraph: The library used to obtain pure graphics in real time, minimizing latency due to plot generation. It is based on the PyQt5 and Numpy libraries.
- Numpy: An important tool for applying numeric and mathematical analysis in various operations inside the algorithm.
- Pandas: Specializes in handling and analyzing data structures in an efficient manner.
- Scipy: Uses its statistical tools to validate results.

The integration of these libraries into the algorithm allowed for the execution of complex functions and the successful estimation of B2B synchrony in real time.

### 2.2. Simultaneous Connection

**Figure 1:**
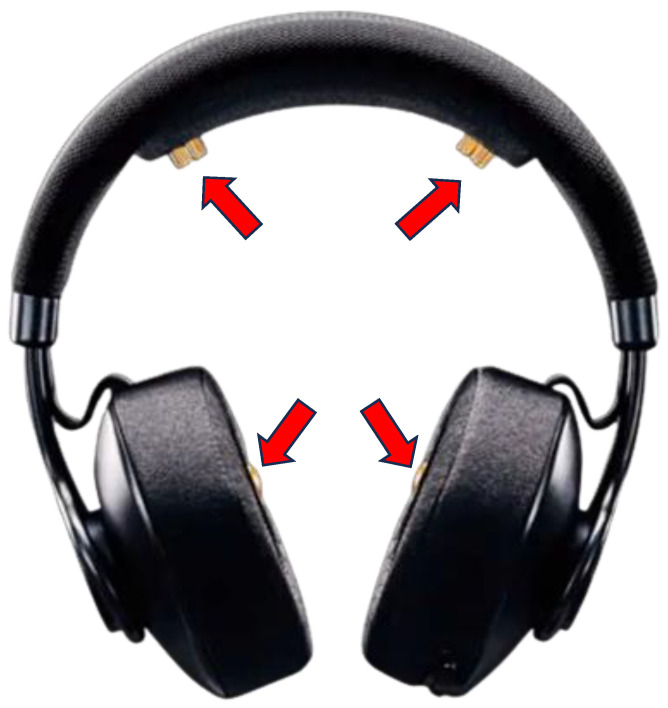
Enophone wearable device used for the development of the real-time B2B synchrony estimation algorithm. Electrodes in this device are marked with arrows.

EEG signal acquisition can be achieved using a variety of commercial devices. To meet the need for a wearable, non-invasive device that can obtain signals in a safe manner, Enophones (Eno, Montreal, CA, USA) were selected. These headphones are equipped with four dry-EEG sensors: two in the top band and two in the ear cups, as shown in Figure 1. This EEG device offers a noise-canceling mode, and each one was purchased for USD 400, making it an affordable option that is highly convenient and practical for carrying out experiments under real-life settings. It has been used in previous investigations to estimate the level of mental fatigue in students using EEG signals and machine learning models, thus functioning in a system that analyzes biometric signals in real time [3].

**Figure 2:**
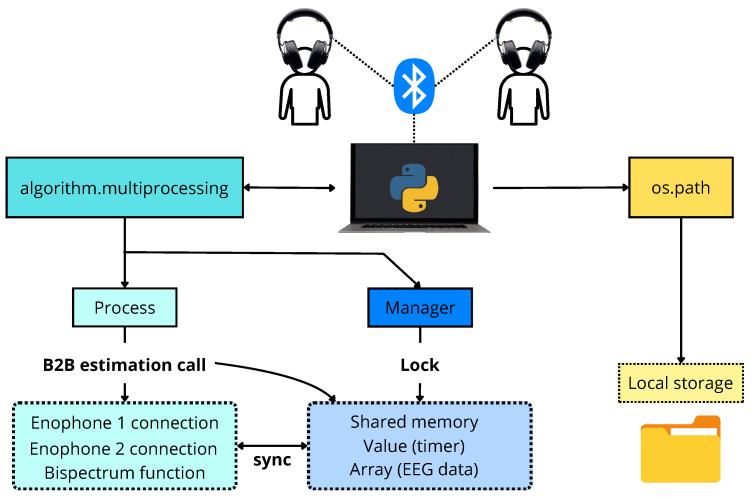
System operation workflow with synchronous mechanism. Two functions, one to establish a connection with each Enophone and another to perform the bispectrum calculation, are created inside the algorithm. Multiprocessing classes (Process and Manager) are used to create shared objects between these functions, executing them in parallel with a synchronized timer. Once the B2B estimation call is made (order to execute the functions), the Lock object blocks the system to only access data being shared simultaneously in the algorithm.

EEG signals were acquired using a sampling rate of 250 Hz via Bluetooth communication. A pair of Enophones was utilized for the real-time B2B synchrony estimation algorithm’s development. The signals were captured using Enophones once they were connected to a single computer device. Each Enophone was connected separately, but their synchrony was ensured by the algorithm’s construction. It is worth noting that this synchrony is achieved through mechanisms built inside the Python multiprocessing module. Simple variables such as seconds running in a timer and complete arrays of information from the processed data are shared between processes. Techniques like locking, Inter-Process Communication (IPC), and atomic operations work to ensure proper synchronization and data integrity [25]. In summary, a function must be created for each device that will be connected, and then all of them run in parallel, allowing for concurrency. Note that the execution of these functions simultaneously prevents delays related to connection problems, as any computational constraint remains equal for all running systems. Emphasizing how this synchronization is efficient, Figure 2 breaks down each step of the global system architecture. When the algorithm calls the functions with the Process method to execute them in parallel, the call is linear. Hence, an overhead not greater than 1 s can occur, depending on the characteristics of the computational device. This initial time window is not considered for the calculations. Once the loop starts, subsequent time windows have proper simultaneous activation. The Lock class enables synchronization abstraction using the time registered on the manager for all functions, ensuring they always start at the same second in the next iteration. The implementation of local execution together with disaggregated data results in good performance that relies only on basic software mechanisms provided by the Python multiprocessing library (communication and synchronization primitives) [25].

**Figure 3:**
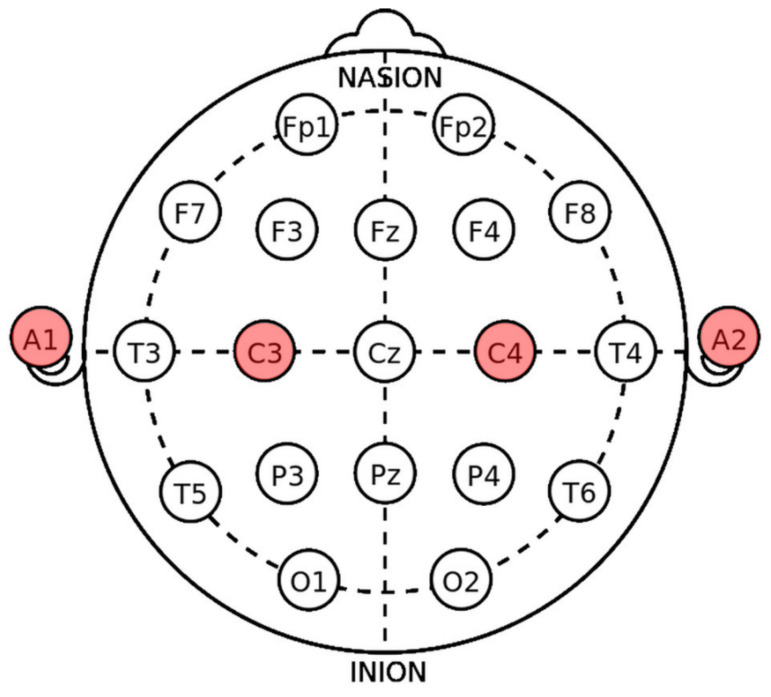
Group of EEG electrodes following the International 10-20 System. Enophones’ electrodes’ positions are shown in color.

In Figure 3, Enophone electrodes are labeled to identify specific channels during experimental tests. The selected letters and numbers correspond to the positions of the electrodes as described by Enophone’s fabricator, following the International 10-20 System.

While the Enophones’ top-band electrodes correspond to C3 and C4 channels, related to the primary motor cortex, research suggests that this region also plays a role in cognitive processes. The dorsal premotor and dorsolateral prefrontal cortices independently manage cognitive operations. The premotor cortex is acknowledged for its role in spatial information processing, while the prefrontal cortex is associated with directing attention to relevant information for executing functions [26]. Research indicates a strong evolutionary correlation between motor cortical areas and the emergence of advanced cognitive abilities, highlighting the crucial role of the motor system in shaping a wide array of cognitive functions seen in both human and nonhuman primates. These functions encompass recognizing and mimicking others’ actions, perceiving and producing speech, as well as executing and appreciating the rhythmic complexities found in music [27]. Despite not being the most suitable regions for cognitive studies, these regions were considered due to their association with various cognitive processes pertinent to the activities being conducted for the validation tests. Furthermore, their selection was influenced by the user-friendly nature of the devices used for signal acquisition.

### 2.3. Signal Pre-Processing

To construct an algorithm for data pre-processing, the first step is to establish a connection with the device that acquires the EEG signal. This connection provides access to raw EEG data, which undergo manipulation through a series of operations and digital filters to yield improved results on denoised (to a certain extent) signals. This initial stage is referred to as pre-processing.

Biosignals are subjected to filtering processes aimed at reducing noise from unwanted sources, and they preserve the content from frequency ranges that contain relevant information [28]. Numerous methods and techniques are available for achieving these objectives. However, the presented algorithm aims to perform all calculations, as well as the B2B synchrony estimations, in real time, which imposes certain computational constraints during the pre-processing stage.

As a result, the pre-processing stage must carefully select filtering methods that strike a balance between noise reduction and computational efficiency. Real-time calculations require filters that are computationally lightweight yet effective in improving signal quality. Additionally, the algorithm may need to prioritize faster processing to ensure timely feedback and response during B2B synchrony assessment. By considering these constraints, the algorithm can achieve real-time data processing while still obtaining reliable and meaningful results from the EEG signals.

Brainflow offers a comprehensive set of functions that encompass a wide variety of pre-processing techniques such as frequency filters, downsampling, and other methods to attain clean EEG signals such as the Independent Component Analysis (ICA) algorithm. In this case, the aim of the algorithm is to perform efficient, real-time pre-processing of two EEG devices simultaneously. Therefore, a custom Python code was created for this purpose, herein limited to a detrending function and the application of frequency (bandpass) filters.

Since the EEG signals are acquired in the time domain, the detrend operation allows us to subtract linear shifts or trends in the data, eliminating distortions caused by the subjects wearing the Enophones and electric drifts. Brainflow provides specific signal processing functions that enable the application of detrend. This serves as the primary safeguard of the algorithm against movement artifacts, long-term noise, and various interferences. Removing linear trends typically does not impact the neural activity of interest, but it may be less beneficial for the non-stationary components of the signal [29]. In addition, to remove noise, a fourth-order Butterworth 0.1–100 Hz Bandpass filter and a 60 Hz Notch filter were implemented. These targeted pre-processing techniques ensure that the algorithm remains computationally efficient and responsive, while still yielding a clean signal suitable for subsequent real-time, B2B synchrony estimation. With this streamlined approach tailored to real-time constraints, the algorithm adeptly handles EEG data processing of two devices simultaneously, allowing for a meaningful and accurate analysis of brain activity in real-time scenarios.

### 2.4. Brain-to-Brain Synchrony Estimation

In the context of B2B synchrony calculations, the bispectrum technique for obtaining valuable information was chosen. Leveraging the Numpy library in Python, the power of the FFT was harnessed to significantly reduce computational demands while maintaining the integrity of spectral component analysis in EEG signals from both subjects.

The use of the multiprocessing module posed a challenge, as it restricts the use of global variables, which complicates data sharing between the functions declared within the algorithm. However, it is worth noting that without this module, none of the previously explained processes (parallel pre-processing and bispectrum estimation) would be feasible.

To address this challenge, the approach turned to shared memory, a feature also available in the multiprocessing module, This technique migrates data among multiple programs; in this case, it helps to provide communication between processes using different virtual addresses for a unique physical location in the memory [30]. It is essential to emphasize that the multiprocessing library parallelizes the execution of algorithm functions, introducing some obstacles. Without delving into the programming specifics of these processes, it suffices to state that in this specific algorithm, matrices generated in the pre-processing stage are decomposed and packaged into shared memory as arrays. The number of arrays corresponds to the number of electrodes compiling information, eight in this case (four from each subject). Once these arrays are imported, they are reconstructed to facilitate the application of the bispectrum function, which iterates over the structure of the original matrix.

The bispectrum was calculated using Equation (1), where $X_{l}$$\left(f_{i}\right)$ and $X_{l}$$\left(f_{j}\right)$ denote the FFT of each time window *l*, using two frequency variables $\left(f_{i},f_{j}\right)$, respectively, and $f_{j}=f_{i}$; $X_{l}^{*}\left(f_{i}+f_{j}\right)$ calculates the conjugate of the FFT of the sum of the frequencies used. Using this equation, the bispectrum is calculated for each 4 s window in real time, for participants *a* and *b*.

(1) $$B{\left(f_{i},f_{j}\right)}_{P_{ab}}=log\left|X_{l}\left(f_{i}\right)X_{l}\left(f_{j}\right)X_{l}^{*}\left(f_{i}+f_{j}\right)\right|$$

Since the experimental protocols involve different pairs of subjects, the bispectrum function calculates the synchronization between each pair of channels between both subjects, resulting in a total of sixteen combinations for this specific case (for a general case: $N^{2}$ combinations, where *N* is the number of EEG channels). The distribution of the bispectrum combinations estimated in this array is shown in Table 1. The reason for using the total of possible combinations is based on the multidimensional nature of the signals. When it comes to hyperscanning, considering all the combinations between two subjects’ neural connections serves to identify paired data with an actual hyper-connection [31]. Moreover, similar studies using a higher number of channels and considering more suitable brain regions state the importance of including all combinations to reveal hidden information in untypical channels and avoid bias [32].

Furthermore, a set of conditions within this function to establish the B2B synchrony levels during a calibration (no-interaction) stage was implemented. This stage allows us to extract data from the first minute of recording, when subjects are in a basal state without interruptions and interactions. The bispectrum data obtained in each window are stored in a CSV file and then averaged across windows. These data serve as a reference for normalizing subsequent activities performed by the subjects in real time. In this sense, the real-time estimations are relative to this basal state and provide a sense of higher or lower B2B synchrony, compared to a no-interaction state.

**Figure 4:**
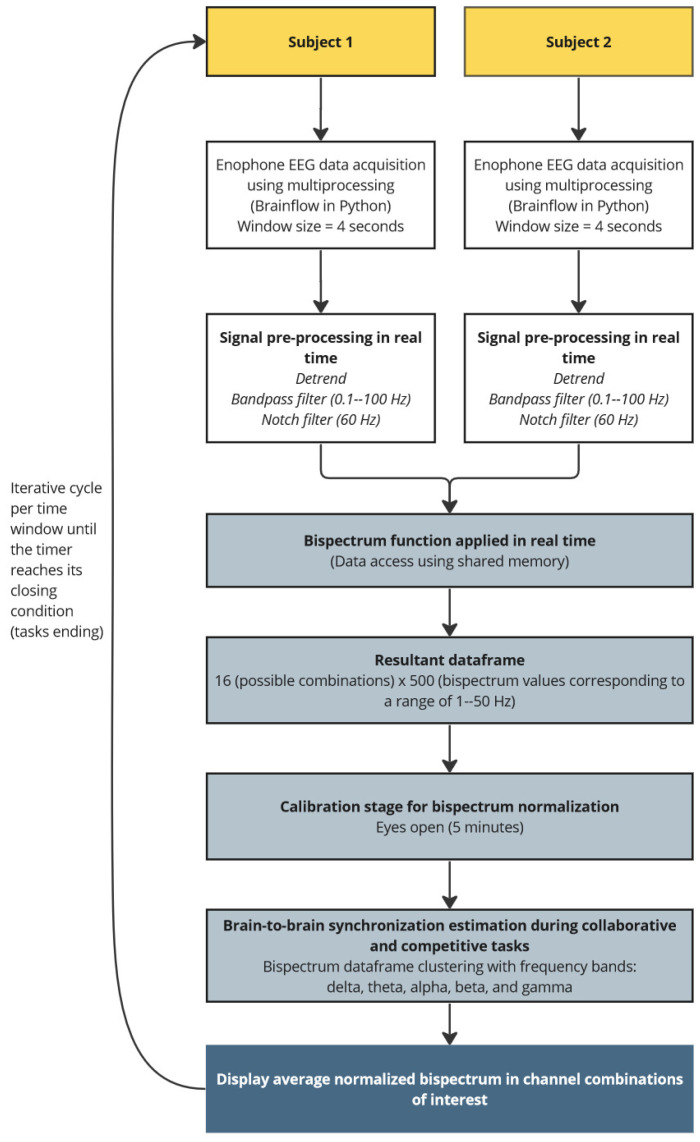
A proposal of the overall methodology that is used as a framework for multiple and simultaneous EEG data collection of two subjects, parallel pre-processing and real-time bispectrum estimation. The bispectrum estimation process is repeated across time windows.

Finally, the resultant bispectrum matrix with normalized values is further interpreted into its EEG frequency components: Delta (0.1–4 Hz), Theta (4–7 Hz), Alpha (8–12 Hz), Beta (13–30 Hz), and Gamma (30–50 Hz), considering the number of (frequency domain) samples obtained per (4 s) time window and the Nyquist frequency (125 Hz) for the 250 Hz sampling rate. The new data array is averaged and displayed for every frequency band, storing it again in another CSV file. The process of real-time estimation of B2B synchrony is shown in Figure 4. A previous study, utilizing the same time window and experimental test duration, was conducted to analyze inter-brain communication between professional musicians during a live jazz performance [4]. This investigation aims to replicate most of the implementations but in real time. The selected duration aligns with an appropriate range of data and allows for quick computing.

### 2.5. Experimental Testing

In order to assess the effectiveness of the proposed algorithm, it could be evaluated under different, contrasting types of activities. For instance, notorious changes in B2B synchrony are observed when participants perform collaborative (higher) and competitive (lower) activities [33].

For the validation process through experimental testing, the participants were recruited through digital platforms and community outreach within the university. The aim was to assemble a diverse group composed of students from different majors to ensure robustness and generality of findings.

A validation was carried out to evaluate the proposed algorithm, using competitive and collaborative activities. In the collaborative problem solving task, participants work together to assemble a puzzle. This task is designed to foster social interactions among participants and encourage them to formulate a strategy to achieve a common objective. On the other hand, the competitive task requires participants to focus on their individual strategies, as they engage in a one-on-one dominoes match.

During the validation of the project, multiple resources were used, such as the following:

- Computing device: A high-performance computing device was implemented to run the algorithm. Within the main features of this computer, it includes an Intel Core i7-9750H CPU @2.60 GHz, 16 GB RAM memory (Intel, Santa Clara, CA, USA), and 64-bit Windows operating system.
- Google Forms: An easy-to-use web application developed by Google for the administration of surveys. Participants were asked to provide demographic information and consent to participate voluntarily in this demonstration.
- Visual Studio Code: A free, multi-platform source code editor developed by Windows (Microsoft, Redmond, WA, USA). This editor was implemented to develop the Python algorithm capable of acquiring brain signals and analyzing them in real time.
- Puzzle: A 500-piece puzzle of THE BATMAN (Novelty, Naucalpan de Juárez, EDOMEX, MX) was used for testing purposes to facilitate collaboration between subjects.
- Dominoes: In order to promote competition between test subjects, a classic 28-piece dominoes game was implemented.

**Figure 5:**
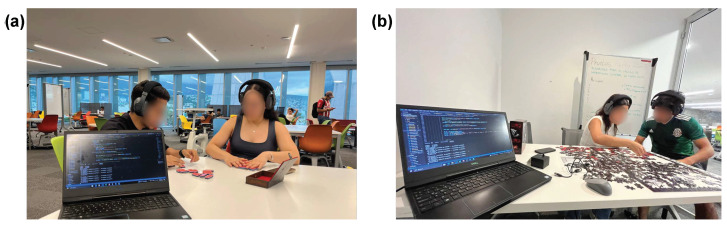
Infrastructure implemented during the performance of (**a**) competitive task (dominoes) and (**b**) collaborative task (puzzle).

To analyze the B2B synchrony during the competitive and collaborative tasks assigned, the procedure presented in Figure 4 was followed. Prior to starting the experiments, a comprehensive explanation regarding the validation tests was provided along with a general review of the algorithm. A complete control of the experiments was performed, giving instructions to the participants to force interaction during collaborative tasks while keeping the noise-canceling mode of the Enophones off. For a standardized procedure, the first task performed by the dyads is the collaborative one, followed by the competitive session. Once the participants had more information about the project and signed the informed consent document, the experiments started making use of the setup shown in Figure 5a,b.

As the aim of this project is to compare inter-brain synchrony values while subjects perform collaborative and competitive tasks, volunteers were asked to perform two 10 min sessions where they played competitive and collaborative games under an in-person modality. Participants were instructed to limit their attention to the task, refraining from communicating verbally if the task was competitive and promoting interaction in collaborative situations.

In these validation tests, eight volunteer couples (sixteen study subjects, aged 21 ± 1 years) participated; each dyad consisted of one male and one female. This grouping selection is influenced by reports suggesting that dyads with at least one male have higher behavioral performance [34]. This is particularly helpful in validating the proposal since higher performance (in a collaborating dyad) has been linked to higher brain synchrony [7]; this in turn is expected to be tracked by the algorithm when comparing the B2B values obtained between (collaboration and competition) groups. Nevertheless, since the algorithm requires wider testing, diverse alternatives of gender grouping have been used in hyperscanning studies [35] and can be applied in future experiments. The subjects were healthy individuals without any mental or physical disorders. The brain signals of the volunteers were measured while performing their assigned tasks, and their B2B estimations were compared. None of the participants were part of the research; however, 3 dyads were formed by volunteers who had theoretical knowledge about EEG but were naive to the experimental procedures.

Participants were asked to remain in a relaxed, no-interaction state with their eyes open for an initial five minutes of the recording to extract their baseline B2B values. The experimental tests started after baseline acquisition. To initiate any chosen activity, it is crucial to engage in it twice consecutively to check results’ repeatability and ensure data reliability. Simultaneously, the recorded data allowed for the calculation of bispectrum values, enabling real-time observation of their behavior.

Studies investigating brain synchrony in both competitive and collaborative scenarios have already been conducted, and similar experimental designs demonstrate that collaborative tasks exhibit higher inter-brain synchrony and show better affinity in results [33,36]. The applied models and architectural foundation of the code were derived from the Advanced Learner Assistance System (ALAS) [3], employing techniques that have undergone statistical validation [4]. The focus is now shifted towards transforming the system into a complete real-time algorithm, enhancing its applicability in educational scenarios, considering that it has been investigated how B2B synchrony is related to students’ class engagement and how it implicates attention mechanisms that can impact teaching effectiveness [12]. However, a more extensive set of experimental tests with a wide number of subjects needs to be conducted, coupled with a more robust offline analysis of the results that will allow us to validate the algorithm’s functionality by itself.

### 2.6. Data Analysis

The framework under which the algorithm was developed allows for the continuous incorporation of new techniques or alternatives to enhance computational efficiency. At critical junctures, raw EEG data were saved to maintain a record of the signal before specific processes were applied. As a result, after real-time experimental testing and validation, a new version was established, significantly improving efficiency and ensuring the successful processing of all data within the specified time windows. To enhance code efficiency and avoid excessive loops, leveraging the Numpy module for vectorization can be a beneficial alternative. However, due to the complexity of operations involving iteration over specific indexes in dictionaries containing data acquired during the calibration stage, it is more straightforward to predefine an array specifying the indexes of interest for each stacked matrix before applying a for loop.

Two of the four electrodes (A1 and A2, as shown in Figure 3) used for the Enophone device are commonly used as reference electrodes. Typical reference strategies entail selecting a reference point either positioned along the midline of the scalp, such as the Cz electrode, or calculating an average reference from both sides of the head (e.g., earlobes) [37]. This feature enhances the discrimination of irrelevant data during signal acquisition. Therefore, a second version of the algorithm includes the A1 and A2 electrodes as references, using the following technique after the pre-processing stage.

(2) $$C3^{\prime }=C3-\left(\left(A1+A2\right)/2\right)$$

(3) $$C4^{\prime }=C4-\left(\left(A1+A2\right)/2\right)$$

Equations (2) and (3) calculate the referenced electrodes’ new values *C*3 and *C*4, respectively, now denoted as *C*$3^{\prime }$ and *C*$4^{\prime }$. Both equations follow electrode labels, as marked in Figure 3. With this new approach, the number of possible combinations changes, as shown in Table 2.

The current version of the algorithm not only enhances efficiency but also affords the flexibility to designate which electrodes should serve as reference points. This feature is useful for fast implementations, as it decreases the computational load of the algorithm by reducing the dimensionality of the real-time estimations.

To validate the results of the eight dyads, an analysis was conducted over all data registered for each frequency band. Given that the algorithm calculates the bispectrum for each time window and improvements are still being developed, a non-parametric statistical analysis is required if sensitivity to outliers is to be avoided. The statistical analysis was performed considering the referenced combinations shown in Table 2. Once the vector was obtained, a Wilcoxon rank sum test was applied by frequency bands to compare the mean normalized bispectrum values for each time window between competition and collaboration. This implementation was carried out subsequently, using the stored data from the experiments and incorporating code utilizing the statistical functions of the Scipy library. The functionality of the code is demonstrated with this implementation. However, as more experimental tests are conducted, involving a higher number of subjects, integrating new types of tasks, and employing deeper statistical analyses, the reliability of the results is expected to increase.

## 3. Results

### 3.1. Algorithm Development

Significant advancements have been achieved in this algorithm, specifically in relation to establishing connections between two Enophones within a single computer device. Four distinct functions were developed within the code, one for each Enophone to establish its connection and pre-process signals individually, but operating in parallel at all times. The two additional functions that run in conjunction with those previously described are those associated with the timer overseeing the algorithm’s duration and the computation of the bispectrum itself. In the initial stages of algorithm development, certain data and timestamps recorded by the manager overseeing both functions were stored in CSV files. This was carried out to verify discrepancies between the acquired signals, which exhibited variations based on the subject’s activity or the connected board.

The algorithm’s structure facilitates the establishment of connections with various devices integrated into Brainflow’s API. Up to this juncture in the development process, no exhaustive verification procedures have been implemented, owing to the straightforward nature of the differences between subjects’ data. However, for subsequent iterations, it would be convenient to consider maintaining timestamp records for each Enophone individually.

The real-time pre-processing stage is limited to the application of digital fourth-order Butterworth filters and a detrend technique to decrease the amount of noise during the signal acquisition process. This stage of the algorithm is integrated inside of the functions that establish the connections with the Enophones.

**Figure 6:**
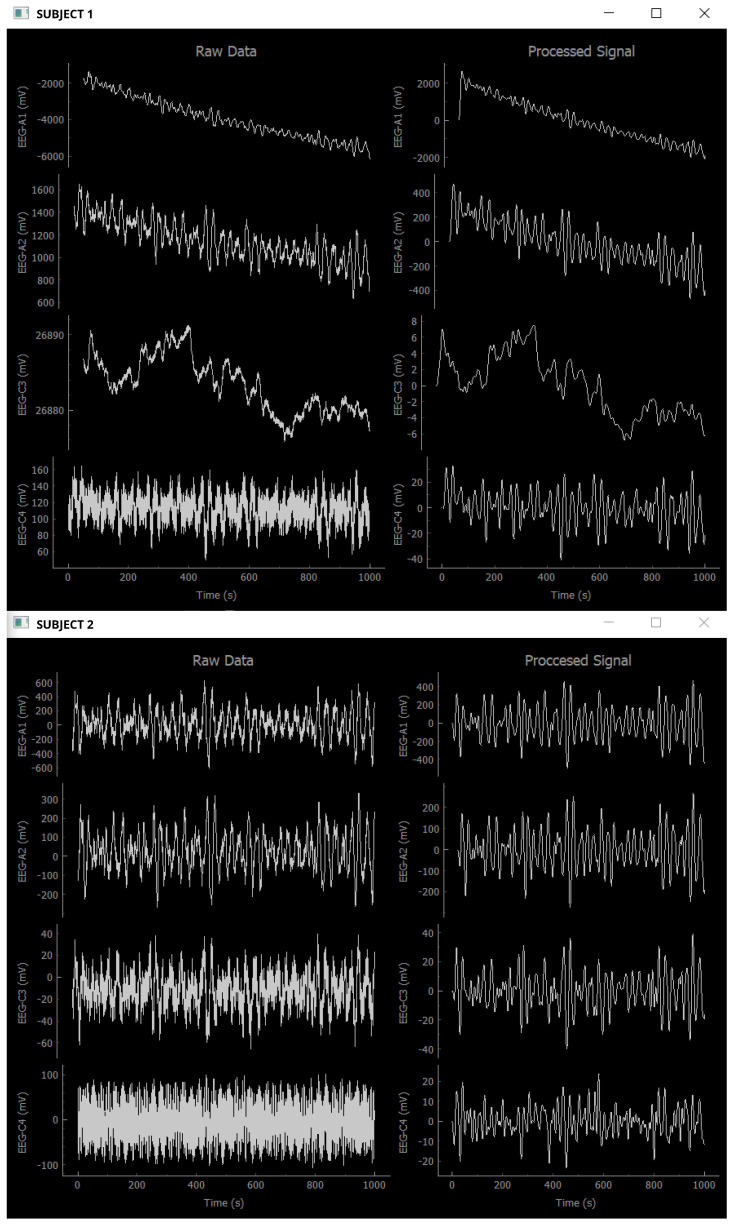
Window display showing the real-time pre-processing stage (right side) to verify filter (Detrend, Notch, and Bandpass) functionality on both subjects (**Top**: Subject 1; **Bottom**: Subject 2). The left side shows the raw EEG of both subjects.

To verify its functioning, the PyQtGraph library was implemented to the environment and raw data were stored apart from the pre-processed data. In this module, the raw and the pre-processed data were displayed side by side for each EEG device. An example of this visualization is shown in Figure 6 for both subjects’ EEG devices. For efficiency purposes, the final version of the algorithm does not include the display of real-time graphics.

While the algorithm is running, the terminal displays normalized bispectrum levels in real time (using 4 s windows), allowing us to monitor how these results fluctuate as subjects engage in various activities. Due to the structure of the code, the bispectrum is stored in a matrix. In this matrix, the columns represent different channel combinations, and the rows group the B2B indicators by the number of samples per time window (e.g., the matrix size is 16 × 500). Additionally, mean bispectrum values (across frequency) are estimated within the range of each frequency band. As a final result, an array that contains numerous bispectrum levels is obtained, corresponding to the combinations generated for each frequency band.

The currently available version of the algorithm has overcome efficiency issues and can now be executed reliably. The implemented upgrades promise improved performance, allowing all data to be interpreted and pass through the constructed processes within the algorithm every 4 s.

For better comprehension, the general algorithm’s architecture is illustrated in Algorithm 1. As depicted in the pseudocode, four functions are created. The first one is the timer controlling the duration of the tests, the second and third functions handle the establishment of connections and data pre-processing, and the fourth function calculates the bispectrum. In this stage, the data from both Enophones are merged, and the estimation of B2B synchronization is normalized. Finally, the main code executes all functions simultaneously. Here, it is evidenced how the creation of shared structures using the multiprocessing module helps incorporate a time variable (second) governing the entire script. More importantly, it establishes a variable of communication (data) between functions, mitigating the risk of race conditions. The folder variable facilitates access to data at specific points in the functions, enabling the storage of data from different devices in different routes. Additionally, it allows for the tracking of the final data output.

### 3.2. Validation

While the current version of the algorithm does not generate visualizations of the filtering process during runtime due to efficiency constraints, it is validated in previous sessions, as it remains essential to have clean signal in the B2B synchrony estimation stage. Alternatively, and computationally lighter, a data display in the terminal can be used to illustrate a successful multi-connection process and how the pre-processing continues to operate individually on each Enophone.

**Figure 7:**
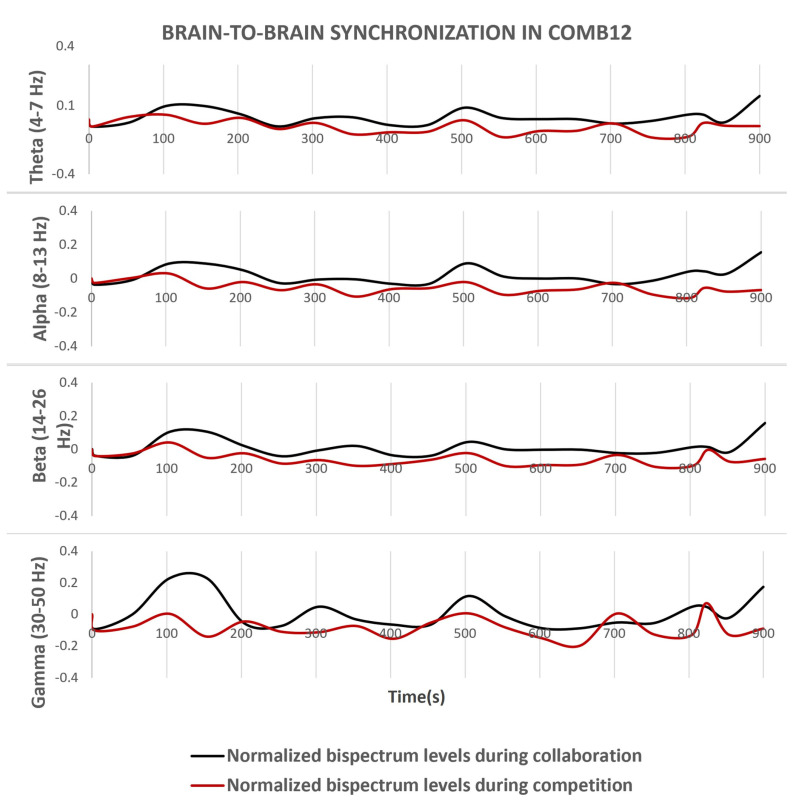
Normalized B2B synchrony (bispectrum) levels between two participants during fifteen minutes of testing, comparing Subject 1 (channel C3) and Subject 2 (channel C4).

**Figure 8:**
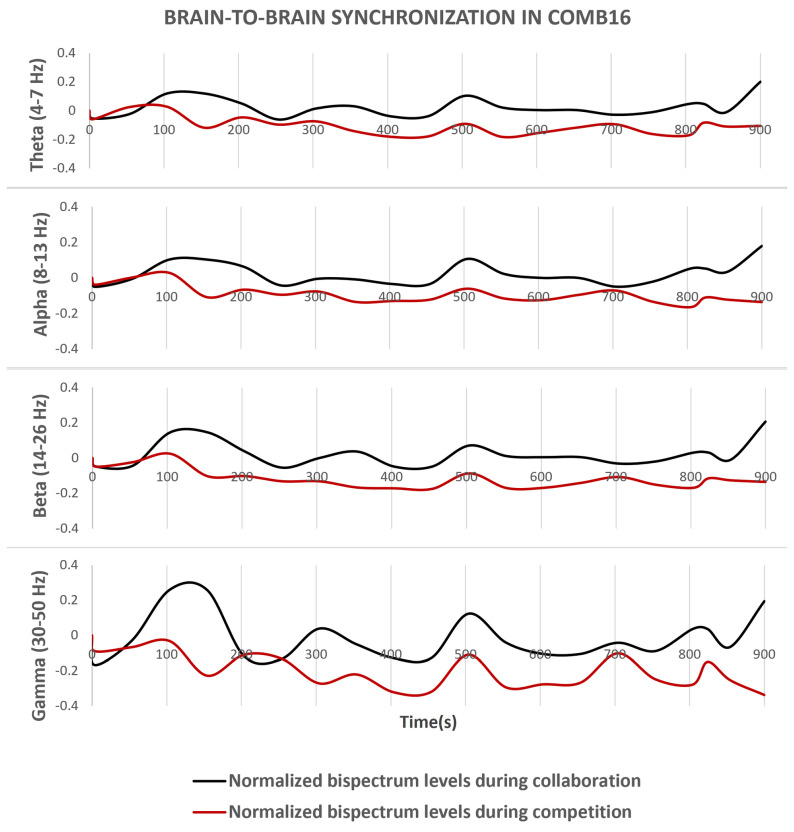
Normalized B2B synchrony (bispectrum) levels between two participants during fifteen minutes of testing, comparing both subjects (channel C3).

Following the pre-processing of signals, the bispectrum function calculates the B2B synchronization levels. In nearly all cases, combinations yielded higher results when subjects engaged in collaborative tasks, compared to competitive tests such as playing dominoes. Figure 7 and Figure 8 depict the fluctuation of the normalized bispectrum relative to the calibration line set at zero. Notably, the bispectrum range expanded in higher frequency bands, particularly during puzzle performance.
**Algorithm 1:** B2B synchrony estimation algorithm
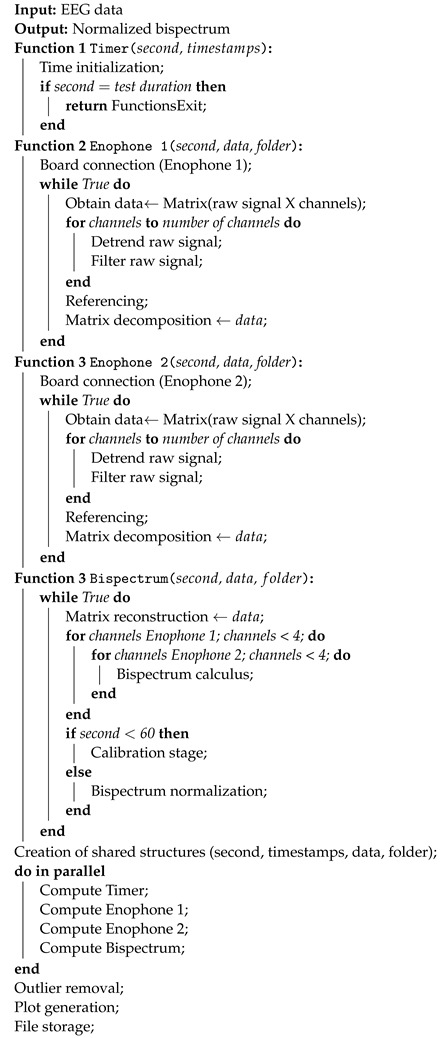


At specific stages of the algorithm, data are saved in a CSV file to maintain a record of the applied changes. The raw and (real-time) pre-processed signals are stored in separate folders, while (real-time) estimated bispectrum results are written to the main folder of the experimental test. Additionally, figures displaying the fluctuation of the bispectrum in each frequency band are stored and presented at the end of the code execution. This methodology enables the extraction of stored data from both collaborative and competitive tasks, facilitating comparison in single graphs, such as the ones shown in Figure 7 and Figure 8.

**Figure 9:**
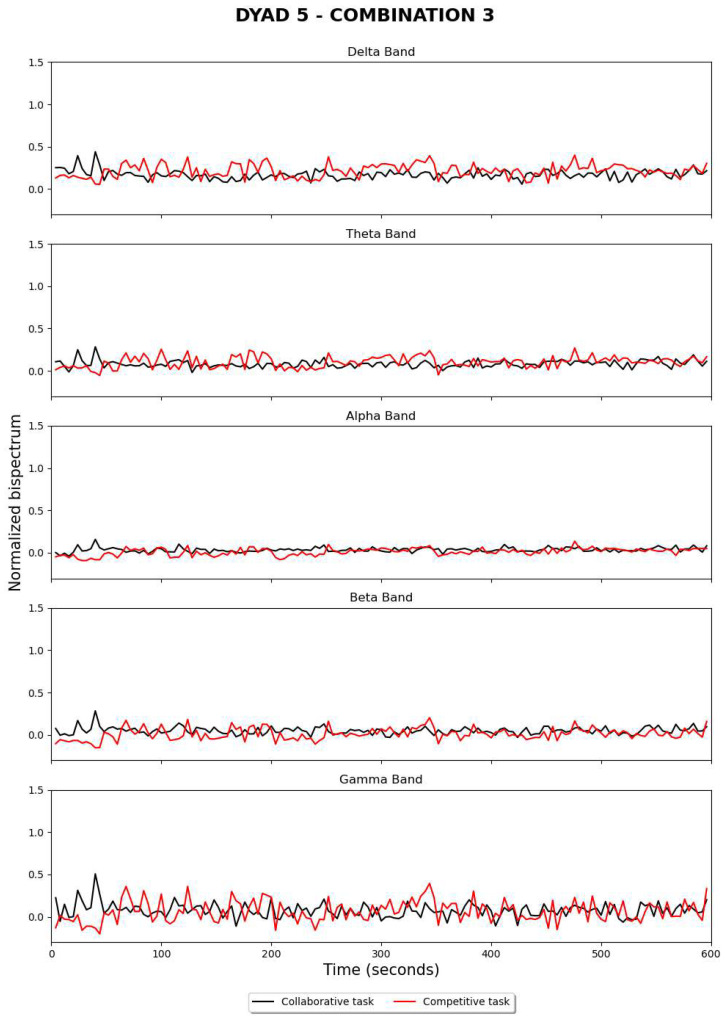
Significant results for dyad 5, using electrode re-referencing during an offline post-analysis.

**Figure 10:**
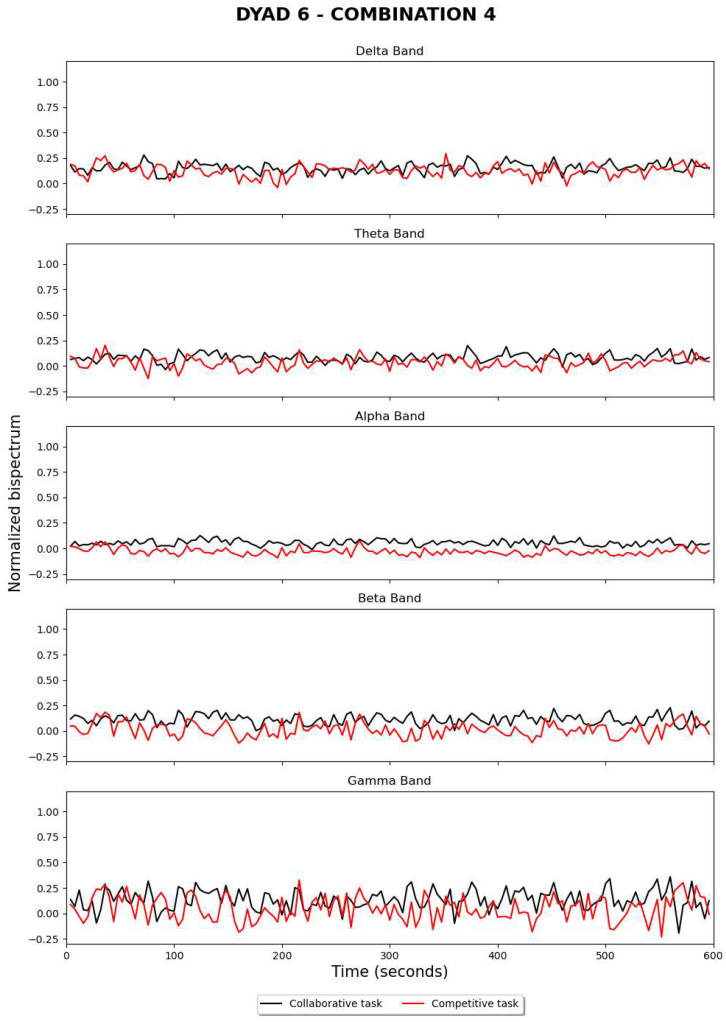
Significant results for dyad 6, using electrode re-referencing during an offline post-analysis.

Figure 9 and Figure 10 show the estimation of bispectrum changes when electrodes are referenced during competitive and collaborative tasks, respectively. This operation was carried out offline using an alternative code that accessed the stored CSV files. The same structure was implemented in the real-time algorithm to display graphs illustrating the fluctuation of the normalized bispectrum over time. The curve exhibits a similar behavior when compared to previous results, but it appears to be more stable.

**Figure 11:**
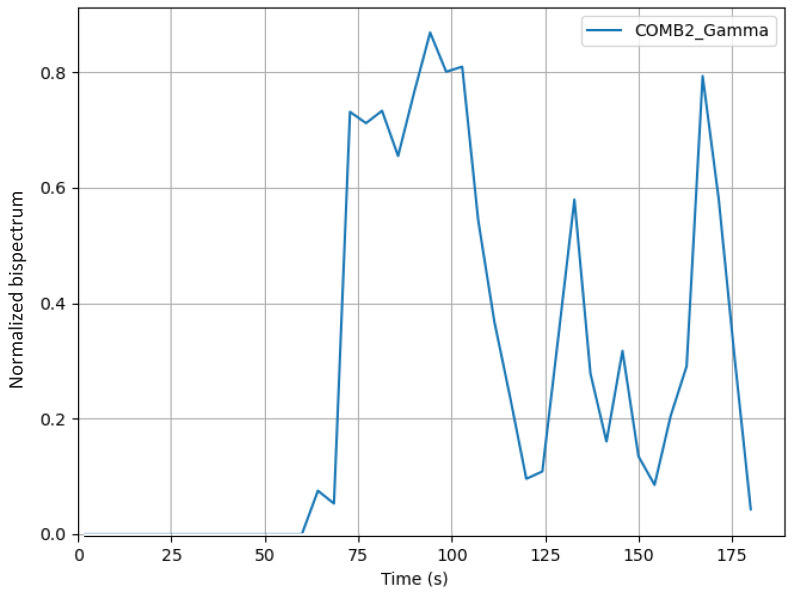
Graph of normalized bispectrum in gamma frequency band using the channel C$4^{\prime }$ subject one with channel C$3^{\prime }$ subject two combination.

Running a verification routine to check the functioning of the code improvement, two subjects attempted to assemble a puzzle for three minutes. In Figure 11, observation reveals that the data become more consistent after the implementation of the optimization. It is important to note that Figure 11 is included solely for illustrative purposes.

In all cases, values above zero indicate a higher level of EEG signal synchrony compared to those recorded during the calibration stage. Consequently, negative results suggest a reduced degree of coupling between subjects, compared to the same stage. Since the results are normalized, the bispectrum will always fall within the range of −1 to 1. Following this approach, peak positive values in the graphs represent the maximum B2B synchronization levels achieved.

**Figure 12:**
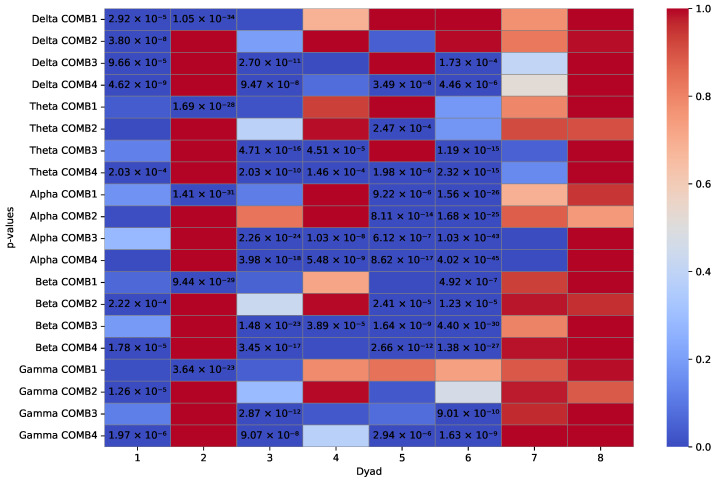
Wilcoxon rank sum (right-tailed) *p*-values. The distribution of bispectrum values underlying collaborative tasks is stochastically greater than the distribution underlying competitive tasks. Numeric significant results are marked for their corresponding dyad and band frequency combinations.

**Figure 13:**
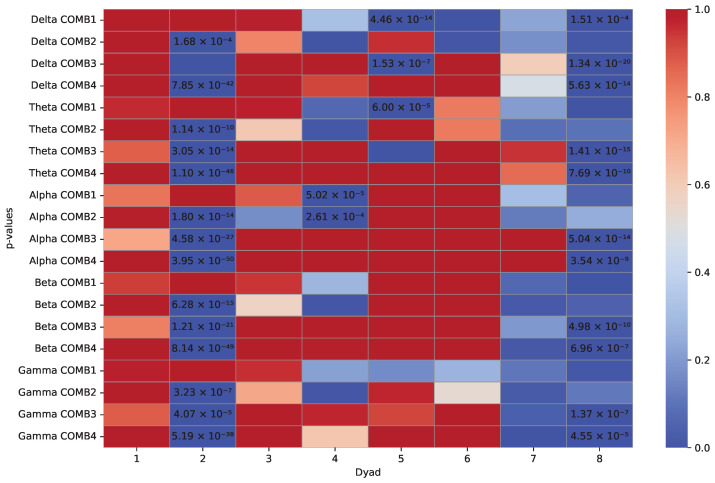
Wilcoxon rank sum (left-tailed) *p*-values. The distribution of bispectrum values underlying collaborative tasks is stochastically lower than the distribution underlying competitive tasks. Numeric significant results are marked for their corresponding dyad and band frequency combinations.

Right-tailed and left-tailed Wilcoxon rank sum tests (160 = 8 dyads × 5 frequency bands × 4 channel combinations) were applied to compare whether bispectrum values in collaboration tasks were higher or lower (respectively) than those in competitive tasks. A total of 33.75% of the right-tailed tests obtained significant *p*-values, while only 18.75% of the tests were significant for the left-tailed test. Significant values for each statistical test are marked as seen in Figure 12 and Figure 13. This analysis demonstrates that the bispectrum values of the first task (collaborative) are higher than those of the second task to which they are being compared (competition) for the majority of dyads. The null hypothesis, stating that there is no difference between the kinds of tasks performed, can be rejected, considering that 54 of the 160 *p*-values are below 0.0003125 (α=0.05 significance level adjusted via Bonferroni correction) in the right-tailed tests. Moreover, only 30 of the 160 *p*-values were significant for left-tailed tests. These results reflect that the real-time estimations of B2B exhibited higher values in the collaborative task, when compared to the competitive task, as has been previously reported by other works [7].

The decision to apply statistical analysis to data processed offline serves as evidence of the reliability of real-time bispectrum results, as demonstrated in Figure 7 and Figure 8. This can be attributed to the use of the same channels (C3 and C4) as depicted in the analyzed figures, along with the referencing improvements made to the electrodes of these channels, exhibiting various normalized bispectrum peak values. The statistical test compares collaborative results against competition, and the support of the *p*-value confirms the existence of statistically significant differences, a behavior prominently reflected in the high-order frequency bands detailed in Figure 12.

## 4. Discussion

The versatility of the proposed algorithm to adapt to different connected boards is a key factor that diversifies its applications and enhances the quality of results. The selection of Enophones was a deliberate decision based on the context of the investigation and the type of validation implemented. This choice was not only due to their safety and non-invasive nature for acquiring EEG signals. Considering the use of headphones capable of recording such signals while subjects are stimulated through sounds, an opportunity to register auditory potential biomarkers is open [38]. This specific trait makes them suitable for educational applications. Moreover, the utilization of Brainflow enables compatibility with a wide variety of devices, opening up possibilities for implementing this framework with other commercial EEG devices.

Another advantage of the algorithm is its ability to connect multiple devices as needed. However, it is important to note that specific functions must be created for each new device to ensure individual processing. This means that declared variables inside these functions work in the same way, but connection parameters are declared individually for each device. To access the complete range of devices available in Brainflow, refer to its Supported Board Documentation “https://brainflow.readthedocs.io/en/stable/SupportedBoards.html (accessed on 20 February 2023)”. General specifications for adjusting Enophones’ connections are described below:

- params: BrainFlowInputParams(), instruction to initialize the connection to any device.
- params.macaddress: “F4:0E:11:75:76:78”, every Enophone possesses a unique identifier with the device it is connected to.
- board: BoardShim(BoardIds.ENOPHONEBOARD, params), a specific BoardIds identification in Brainflow needs to be written if another kind of device wants to be connected.

Utilizing the multiprocessing library, it becomes possible to execute all functions defined within the algorithm in parallel for each acquisition device. Using specialized functions provided by this library allows us to concurrently operate all the code, ensuring its functioning under a manager that keeps track of the code execution in seconds. However, implementing timestamps individually for each connected device is a valuable strategy for verifying that data recording and processing are synchronized accurately between both Enophones. Brainflow offers the capacity to store the timestamps of the Enophones by itself; currently, none of this information is exploited in the algorithm, but in posterior offline data curation, the synchrony was confirmed by the register of those timestamps. It could be important to mention that in certain time windows, the difference between the timestamps for the first data values of Enophone 1 and Enophone 2 was that of milliseconds. A complete and exact parallel execution with no differences can be achieved if the barrier mechanism is employed [25]. Note that data acquisition is ruled by the time in the manager that is actually synchronized in all functions, but this implementation can serve as a higher and automated verification method.

The characteristics of the pre-processing stage can be adjusted to improve results, but it is crucial to consider that all of the algorithm’s operations should yield real-time interpretations, graphs, or results. Therefore, any modifications must be made with computational constraints in mind. Utilizing higher-order filters or robust model analysis should enhance the algorithm, and Brainflow provides the tools to achieve this. Infinite impulse response (IIR) filters are the best option for good frequency response feedback, but phase distortion can be a problem as the order keeps growing. However, in all cases, it is important to assess whether these tools can be implemented without damaging the efficiency of real-time B2B synchrony estimation. The modules implemented to graph results handle large numbers of data efficiently. Given their resource-intensive characteristics, it is advisable to present results in simpler formats. Once the algorithm’s efficiency is optimized and all the previously discussed aspects are working perfectly, optimized visualizations can be explored to represent real-time estimations.

The bispectrum technique used in this algorithm utilizes data extracted within the specified time windows, resulting in two-dimensional matrices that contain the brain synchrony of signals across all frequency bands. This encompasses all possible combinations of electrodes between both subjects using the Enophones. While it may not be possible to provide a deeper evaluation by compiling data and iterating between time windows, as is performed in offline bispectral analysis [18], the results displayed thus far suggest that analyzing each window individually provides valuable information to obtain appropriate insights into B2B synchronization.

During the validation process, notable differences were observed among various types of activities. Specifically, higher normalized bispectrum results were observed when subjects were collaborating, particularly in higher frequency bands. There are several reports in the literature that point to higher neural synchrony during these types of interactions [36]. This suggests that social interactions during collaborative activities requiring the use of certain cognitive processes engage subjects in synchronization more effectively than competitive tasks. Moreover, the fact that the proposed algorithm was able to capture higher B2B synchrony in the collaboration task during the validation stage is an encouraging result. The straightforward statistical implementation of Wilcoxon rank sum tests, comparing data between different activities, contributes to demonstrating the reliability of the algorithm, aligning with studies reporting higher brain synchrony during collaboration [7,33]. It is intriguing to observe that the collaboration task exhibits higher normalized bispectrum values in mid- and high-order frequency bands, along with significant *p*-values for a great number of combinations. This observation aligns with a higher synchrony as participants interact. However, it is crucial to note that the sample size of the study remains small. The significant values just confirmed the algorithm’s functioning, marking its ability to estimate B2B synchronization and yield effective analysis as other studies; nevertheless, even with a greater percentage of significant *p*-values in the Wilcoxon right-tailed test, specific dyads shows a contrary behavior, which restricts a forceful conclusion. This kind of problem can be related to insufficient signal pre-processing and data outliers when subjects do not engage in the activity as they are supposed to, since all the analysis is applied in the short term due to real-time applications. While the current results support the functioning of the algorithm, for a more extensive validation, a significant number of subjects should be evaluated to avoid estimation bias; moreover, increasing the quantity of persons being analyzed can be an option if alternative techniques are employed to study collective social interactions.

The latest version of the algorithm includes efficiency improvements. However, the validation routine with a new group of subjects remains pending. The development of this proposed algorithm represents a significant contribution to the field of neuroengineering. Another advantage of this algorithm is that it offers an open-access code that is easily implementable on any operating system and is accessible to institutions or companies seeking to benefit from tools that can estimate levels of synchronization in humans.

The field of BCI research impacts different domains, including human behavioral interaction, education, and cognitive behavior analysis. Various studies exemplify the measurement of brain synchronization among individuals using different methodologies. The contrast between previously developed algorithms and the one presented in this study lies in the capability to connect various EEG devices for signal acquisition and data processing with feature extractions in real time. The basis of the implemented bispectral analysis was used to study social interactions and inter-brain communication in collaborative settings, as exemplified in the study “Brain-to-brain communication during musical improvisation: a performance case study” [4].

The development and implementation of this algorithm directly impact the fields of hyperscanning and neuroeducation. On one hand, the analysis is conducted through the interaction of two individuals simultaneously, guiding their social interactions to determine how specific activities impact levels of interconnection. Moreover, a set of conditions in the experimental phase was planned to involve subjects in activities requiring high cognitive processes such as memory and attention, cognitive processes intricately linked to learning and, therefore, to educational contexts [9].

As this type of algorithm becomes more frequently implemented in real-life scenarios, B2B synchronization research will increase, as well as its applications and reach. Hyperscanning metrics offer valuable insights for studying brain behavior, and in the educational context, they can be used to provide feedback on teaching and learning performance. It is important to address that this type of algorithm has the potential to be valuable not only for neuroeducation but also in any contexts involving interactions with instructional purposes, such as marketing and communication [39].

## 5. Conclusions

Having access to real-time feedback on situations where subjects demonstrate higher performance in activities requiring social interactions and high cognitive processes can be a valuable tool in educational institutions [40]. It enables the generation of immediate adjustments to teaching methods and provides continuous monitoring of the learning process.

B2B synchronization plays a crucial role in education, as the interaction between students and teachers directly influences brain activity and cognitive performance [41]. In the context of education, various teaching models have been developed to test new learning techniques. However, the evaluation of learning model optimization often depends on individual diagnostics, and a lack of effective communication between neuroscientists and educators results in slow, gradual growth [9]. Currently, a significant gap exists in terms of specific techniques or technologies that can evaluate the impact on learning levels by considering real-time physiological changes occurring in the brain [7]. Therefore, the discoveries made in this study hold immense value as they present an opportunity to not only enhance the efficiency of the algorithm but also provide a solution to the previously identified problems. Moreover, these findings can potentially be applied within a classroom setting during regular classes [12].

Creating a real-time algorithm is a complex task that requires considerable computational power. As noted throughout this work, multiple strategies have been approached to perform the desired functions using custom codes. Even with the current efficiency of the algorithm, further work is essential in order to explore alternative programming techniques to ensure more optimal performance and to allow the incorporation of more complex processes and functions into the algorithm, such as graph visualization and the full potential of reducing the execution time of the multiprocessing module [25]. All of these issues will be addressed in the next steps of this implementation. As the algorithm consistently includes a calibration stage during which subjects are in a no-interaction state to normalize the bispectrum, it becomes evident that interactions or tasks demanding the use of shared cognitive processes lead to an increase in bispectrum levels. This observation underscores the algorithm’s potential as a reliable metric that can find applications not only in classrooms but also in various industries for tracking the effectiveness of training courses or situations involving social interactions between at least two individuals, taking into account that elevated levels of B2B synchronization may result in enhanced communication and performance among subjects [42].

Further conditions need investigation, and new types of activities and tasks can be introduced and evaluated. While the results obtained so far demonstrate the algorithm’s capability to calculate the bispectrum in real time for pairs of subjects, it is important to conduct a more robust statistical analysis, comparing data with well-established procedures in offline analysis of this technique [4] to assess its precision. Significant *p*-values found in collaborative tasks serve as evidence of the algorithm’s capability to distinguish B2B synchronization in different contexts. A total of 33.75% of significant results in collaborative activities can be considered a low estimation, but considering that competition exhibits an even lower significance (18.75%), it is important to remark upon the sufficient evidence to coincide with other studies reporting higher inter-brain synchrony during collaboration [7]. More importantly, this helps to construct the basis for a functional algorithm with real-time B2B synchrony estimation capacities. Its implementation is expected to foster the development of new studies using real-time metrics in various scenarios that involve group activities, similar to work–life situations, to deepen the understanding of tasks requiring social interactions.

## Figures and Tables

**Table 1:** Index combinations (COMBs) used to calculate the bispectrum between EEG signals acquired from every channel located in a set of two 4-channel Enophones.

COMB	Subject 1	Subject 2
1	A1	A1
2	A1	A2
3	A1	C3
4	A1	C4
5	A2	A1
6	A2	A2
7	A2	C3
8	A2	C4
9	C3	A1
10	C3	A2
11	C3	C3
12	C3	C4
13	C4	A1
14	C4	A2
15	C4	C3
16	C4	C4

**Table 2:** Index combinations (COMBs) considering just referenced electrodes.

COMB	Subject 1	Subject 2
1-ref	C$3^{\prime }$	C$3^{\prime }$
2-ref	C$3^{\prime }$	C$4^{\prime }$
3-ref	C$4^{\prime }$	C$3^{\prime }$
4-ref	C$4^{\prime }$	C$4^{\prime }$

## Data Availability

The datasets generated and analyzed for this study along with the algorithm implemented can be found in the REAL-TIME B2B GitHub repository “https://github.com/Amisaday74/REAL-TIME-B2B- (accessed on 10 October 2023)”.

## Abbreviations

The following abbreviations are used in this manuscript:

ALAS	Advanced Learner Assistance System
BCI	Brain–Computer Interface
B2B	brain-to-brain
EEG	electroencephalography
FFT	Fast Fourier Transform
ICA	Independent Component Analysis
IPC	Inter-Process Communication
